# Supplementary material for: Contrasting effects on deep convective clouds by different types of aerosols
Source: Nat Commun. 2018 Sep 24;9:3874. doi: 10.1038/s41467-018-06280-4 (PMC6155150; doi:10.1038/s41467-018-06280-4)
Supplement: Supplementary file 2 — Supplementary Information [file 41467_2018_6280_MOESM2_ESM.docx]

**Supplementary**

**Supplementary Figure 1:** The Climatological monthly mean of AOT for smoke aerosol (gray line), dust aerosol (blue line), and polluted continental (P_cont) aerosol (red line) over the three study regions: (top) South America, (middle) Central Africa, and (bottom) Southeast Asia. Solid line represents the AOT in the lower troposphere (> 680 hPa), and dotted line represents the aerosol occurrence frequency in the middle troposphere (440-680 hPa). The error bars indicate ±1 standard deviation of the monthly mean AOT for each aerosol type.

**Supplementary Figure 2:** Annual average changes of the altitude centroid for deep convective clouds (ΔZ_IWC_), computed as the differences of Z_IWC_ between the clean environment and the smoke (gray), dust (blue), polluted continental aerosol (red) environments for different regions and at different AOT ranges: (a) AOT = 0.02 to 0.2; (b) AOT > 0.2; and (c) All AOTs. Note (c) is the same as Figure 1 in the main text. The error bars denote the standard errors of the average

**Supplementary Figure 3**: Seasonal average changes of the altitude centroid for deep convective clouds, computed as Z_IWC_ differences between clean environment and different aerosol environments: smoke aerosol (gray), dust aerosol (blue) and polluted continental aerosol (red) over the selected three regions: (left) South America, (middle) Central Africa, and (right) Southeast Asia. All AOT values are included for this plot. The error bars denote the standard errors of the average.

**Supplementary Table 1:** Total correlations between column AOT and IWC centroid, and the partial correlations with the effects of 12 meteorological parameters eliminated individually and simultaneously. AOT range is [0,0.25]. Bold font indicates the significant agreement (same sign) between total and partial correlation, whereas non-bold font indicate the significant opposite signs between them. If total/partial correlation is not statistically significant at the 95% level, the corresponding font is italic.

|  |  | **Winter (DJF)** | | | **Spring (MAM)** | | | **Summer (JJA)** | | | **Fall (SON)** | | |
| --- | --- | --- | --- | --- | --- | --- | --- | --- | --- | --- | --- | --- | --- |
|  |  | **smoke** | **dust** | **polluted continental** | **smoke** | **dust** | **polluted continental** | **smoke** | **dust** | **polluted continental** | **smoke** | **dust** | **polluted continental** |
| **South America** | **Total correlation** | **–0.13** | **–0.072** | **0.095** | *0.000* | **0.044** | **0.25** | **–0.12** | **0.24** | **0.24** | **–0.069** | **0.067** | **0.12** |
|  | RH_850_ | **–0.12** | **–0.068** | **0.11** | *-0.027* | **0.040** | **0.26** | **–0.12** | **0.23** | **0.24** | **–0.064** | **0.060** | **0.12** |
|  | RH_500_ | **–0.12** | **–0.068** | **0.11** | *-0.027* | **0.040** | **0.26** | **–0.12** | **0.23** | **0.24** | **–0.064** | **0.060** | **0.12** |
|  | RH_350_ | **–0.13** | **–0.083** | **0.079** | *-0.036* | *0.037* | **0.26** | *–0.069* | **0.23** | **0.24** | *–0.040* | **0.078** | **0.16** |
|  | LTS | **–0.13** | **–0.072** | **0.099** | *-0.041* | **0.047** | **0.23** | **–0.14** | **0.25** | **0.24** | **–0.061** | **0.061** | **0.13** |
|  | VV_500_ | **–0.13** | **–0.066** | **0.098** | *-0.015* | *0.032* | **0.24** | **–0.15** | **0.18** | **0.21** | **–0.082** | **0.048** | **0.094** |
|  | VV_300_ | **–0.13** | **–0.060** | **0.10** | *0.013* | **0.043** | **0.26** | **–0.16** | **0.15** | **0.22** | **–0.078** | **0.058** | **0.11** |
|  | U_300_ | **–0.14** | **–0.076** | **0.096** | *-0.027* | **0.008** | **0.24** | **–0.13** | *0.052* | **0.18** | **–0.088** | **0.076** | *0.021* |
|  | U_1000_ | **–0.10** | **–0.073** | **0.10** | *0.032* | **0.045** | **0.26** | **–0.11** | **0.23** | **0.23** | **–0.093** | **0.072** | **0.13** |
|  | V_300_ | **–0.13** | **–0.077** | **0.091** | *-0.014* | **0.042** | **0.27** | **–0.14** | **0.17** | **0.22** | **–0.097** | **0.047** | **0.10** |
|  | V_1000_ | **–0.13** | **–0.076** | **0.090** | *0.002* | **0.051** | **0.25** | **–0.12** | **0.24** | **0.25** | **–0.069** | **0.067** | **0.12** |
|  | CAPE | **–0.07** | **–0.070** | **0.064** | *0.029* | *-0.018* | **0.28** | **–0.19** | **0.19** | **0.17** | **–0.069** | **0.009** | **0.13** |
|  | VWSH | **–0.14** | **–0.070** | **0.095** | *-0.005* | **0.047** | **0.26** | **–0.12** | **0.24** | **0.24** | **–0.091** | **0.072** | **0.11** |
|  | **all parameters** | 0.027 | **–0.066** | **0.058** | *0.012* | *0.006* | **0.29** | **–0.15** | **0.15** | **0.12** | **–0.13** | –0.043 | **0.075** |
| **Central Africa** | **Total correlation** | **–0.13** | *0.016* | **–0.11** | *0.032* | **–0.11** | *0.020* | *0.014* | **–0.08** | **–0.065** | **0.091** | **0.098** | **0.17** |
|  | RH_850_ | **–0.13** | *0.017* | **–0.16** | *0.030* | **–0.12** | *0.022* | *–0.027* | **–0.07** | *–0.011* | **0.085** | **0.095** | **0.18** |
|  | RH_500_ | **–0.13** | *0.017* | **–0.16** | *0.030* | **–0.12** | *0.022* | *–0.027* | **–0.07** | *–0.011* | **0.085** | **0.095** | **0.18** |
|  | RH_350_ | **–0.13** | *0.003* | **–0.12** | *0.031* | **–0.13** | *0.016* | –0.076 | **–0.10** | **–0.060** | **0.079** | **0.10** | **0.17** |
|  | LTS | **–0.13** | *–0.002* | **–0.12** | *0.013* | **–0.11** | *0.000* | *0.024* | **–0.06** | *–0.032* | **0.093** | **0.064** | **0.13** |
|  | VV_500_ | **–0.13** | *0.014* | **–0.11** | *0.044* | **–0.12** | *0.026* | *0.011* | **–0.07** | **–0.066** | **0.089** | **0.10** | **0.17** |
|  | VV_300_ | **–0.13** | *0.015* | **–0.11** | *0.020* | **–0.12** | *0.016* | *0.001* | **–0.08** | **–0.059** | **0.086** | **0.099** | **0.17** |
|  | U_300_ | **–0.15** | *0.009* | **–0.13** | *0.017* | **–0.11** | *0.009* | *0.020* | **–0.08** | **–0.066** | **0.092** | **0.098** | **0.17** |
|  | U_1000_ | **–0.14** | *0.005* | **–0.13** | *0.028* | **–0.11** | *0.020* | *0.019* | **–0.07** | **–0.066** | **0.098** | **0.098** | **0.20** |
|  | V_300_ | **–0.13** | *0.024* | **–0.11** | *0.033* | **–0.11** | *0.020* | *0.024* | **–0.06** | ***–0.055*** | **0.091** | **0.098** | **0.17** |
|  | V_1000_ | **–0.14** | *0.011* | **–0.11** | *0.015* | **–0.13** | *0.012* | *0.034* | **–0.08** | **–0.063** | **0.11** | **0.12** | **0.19** |
|  | CAPE | **–0.11** | *0.022* | **–0.11** | *0.036* | **–0.07** | *0.019* | –0.10 | **–0.06** | *0.017* | **0.12** | **0.097** | **0.16** |
|  | VWSH | **–0.13** | *0.018* | **–0.11** | *0.036* | **–0.12** | *0.023* | *0.014* | **–0.08** | **–0.060** | **0.091** | **0.097** | **0.17** |
|  | **all parameters** | **–0.13** | *–0.004* | **–0.15** | *0.008* | **–0.12** | *–0.001* | *–0.008* | **–0.05** | *0.023* | **0.14** | **0.091** | **0.18** |
| **Southeast Asia** | **Total correlation** | **–0.10** | *–0.050* | *0.035* | *–0.019* | **–0.08** | **0.15** | –0.026 | **–0.16** | **0.072** | *–0.029* | **–0.15** | *0.027* |
|  | RH_850_ | **–0.15** | **–0.094** | *0.019* | *–0.026* | **–0.09** | **0.15** | *–0.016* | **–0.16** | **0.065** | **–0.045** | **–0.15** | *0.032* |
|  | RH_500_ | **–0.15** | **–0.094** | *0.019* | *–0.026* | **–0.09** | **0.15** | *–0.016* | **–0.16** | **0.065** | **–0.045** | **–0.15** | *0.032* |
|  | RH_350_ | **–0.096** | *–0.040* | *0.042* | *–0.037* | **–0.09** | **0.18** | **–0.052** | **–0.17** | **0.055** | *–0.032* | **–0.15** | *0.030* |
|  | LTS | **–0.085** | *–0.049* | *0.036* | *–0.036* | **–0.09** | **0.15** | *–0.015* | **–0.16** | **0.065** | **–0.041** | **–0.15** | *0.032* |
|  | VV_500_ | **–0.070** | **–0.12** | *0.005* | **–0.045** | **–0.11** | **0.15** | **–0.035** | **–0.17** | **0.058** | *–0.012* | **–0.15** | **0.034** |
|  | VV_300_ | **–0.10** | **–0.073** | *0.033* | –*0.029* | **–0.10** | **0.15** | **–0.036** | **–0.17** | **0.061** | *–0.010* | **–0.12** | **0.048** |
|  | U_300_ | *–0.052* | **–0.14** | **0.080** | **–0.060** | **–0.15** | **0.17** | **–0.065** | **–0.20** | **0.068** | **–0.068** | **–0.19** | *0.023* |
|  | U_1000_ | **–0.10** | *–0.054* | *0.035* | *–0.009* | **–0.11** | **0.17** | **–0.031** | **–0.15** | **0.093** | *–0.028* | **–0.15** | *0.029* |
|  | V_300_ | *–0.021* | **–0.064** | *0.044* | **–0.10** | **–0.14** | **0.18** | **–0.036** | **–0.16** | **0.072** | **–0.047** | **–0.14** | *0.034* |
|  | V_1000_ | **–0.11** | *–0.047* | *0.036* | *–0.041* | **–0.11** | **0.18** | *–0.020* | **–0.16** | **0.079** | *–0.030* | **–0.15** | *0.028* |
|  | CAPE | **–0.068** | **–0.18** | *0.005* | **–0.11** | **–0.15** | **0.14** | **–0.055** | **–0.19** | **0.092** | **–0.080** | **–0.20** | *–0.002* |
|  | VWSH | **–0.10** | *–0.050* | *0.033* | *–0.016* | **–0.09** | **0.15** | *–0.024* | **–0.16** | **0.072** | *–0.030* | **–0.15** | *0.027* |
|  | **all parameters** | *–0.003* | **–0.16** | *0.041* | **–0.11** | **–0.19** | **0.20** | **–0.065** | **–0.21** | **0.054** | **–0.10** | **–0.16** | *0.025* |
